# Some Dottings on Hemorrhage

**Published:** 1897-04

**Authors:** J. McFadden Gaston

**Affiliations:** Atlanta, Ga.


### SOME DOTTINGS ON HEMORRHAGE.

BY J. McFADDEN GASTON, M. D., ATLANTA, GA.

Hemorrhage is a flow of blood from a blood-vessel by incision, rupture or other solution of continuity.

It may be active or passive; traumatic or idiophatic (bleeders); primary or secondary, venous or arterial, epistaxis, hemoptysis, hemetemesis, hematuria.

Intestinal or rectal hemorrhage uterine hemorrhage, metorrhagia, menorrhagia.

Scurvy with sanguineous exudate from gums. Rupture or ulceration of varicose veins of leg.

Umbilical hemorrhage in infants.

Local use of hot water with vinegar stops oozing of blood. Alum and tannin with compress of cotton. Perchloride of iron. Actual cautery at black heat applied lightly to surface. Use of suture with needles placed at part transversely and longitudinally with figure of eight over each and then encircling all the tissue. The application of plaster of paris in bleeding of gums after extraction of molar tooth has been found to arrest it. Torsion of small twigs of arteries proves effectual, and has been employed successfully in arteries of considerable size by Bryant of England.

Ligature secured in the wound or in the continuity of the vessel on the cardiac portion is safer practice. Catgut is available for deep-seated vessels and admits of immediate closure of the wound. Veins near the skin may be closed by pins and thread, as hair-lip sometimes.

Dr. E. A. Wright in the *Bristol Medical Journal*, 1891, states that a certain per cent. of calcium salts is necessary for coagulation of blood and shows the virtue of chloride of calcium in increasing the coagulability of blood. Reports four cases, one being a child of four years with injury of frenum of upper lip in which it was effective.

Bryant reports case of child four and a half years with a wound above the orbit in which it was used with effect after failure of other means, he gave the chloride of calcium in 1 30 grain doses every four hours first, every six hours in the second, and three times in the third day.

Dr. J. Clifford Perry, Assistant Surgeon U. S. Marine Hospital Service, reports case of a Russian twenty years of age in which a slight incision of the gums led to profuse hemorrhage which resisted ordinary treatment with styptics of perchloride of iron and finally bleeding of the nose occurred but was at last relieved by oil of turpentine, sulphate of soda, and chloride of calcium.

J. Reverdin of Geneva has used successfully sulphate of soda in doses of 1½ grains every hour in grave capillary hemorrhage, spontaneous or traumatic. Given to animals or by intravenous injection it forms coagulation of blood; has been used in hemophelia. Hemorrhage may occur from veins or arteries and from a single vessel or a large number, causing a general oozing from the surface.

The means of arresting hemorrhage are vital, chemico-vital, and mechanical. Vital may operate generally or locally. Cold, heat, alcohol, spirits turpentine, acid, carbolic; internally, ergot, acetate of lead, sulphpric acid, oil of erigeron.

Chemico-vital: tannin, alum, matico, salts of iron, nitrate of silver, and chloride of zinc, acetic acid (vinegar.)

Mechanical means are pressure, position, cauterizing with hot iron, torsion, acupressure and ligature; the fingers, the tourniquet, and various compresses, handle of key, nasal canula for plugging; the forceps for temporary stoppage of bleeding; the use of two forceps for torsion and the forceps of Bryant.

Arteries—version and the slitting with passage of end through the opening so as to occlude the canal.

Acupressure by Sir James Simpson of Edinburgh with iron wire. Plans of Professor Pirrie of Aberdeen University: circumclusion, torsoclusion, retroclusion.

Buck's method of torsion and transfixion is sometimes useful. Hutchinson's plan of exposing the vessel and passing a needle beneath with a loop of wire to secure it, may be employed. A canula with a loop of wire. Screw hook of Spier, Brooklyn City Hospital. Grip forceps for deep vessels; serres-fines for small arteries.

